# Overexpression of Nuclear Protein Kinase CK2 α Catalytic Subunit (CK2α) as a Poor Prognosticator in Human Colorectal Cancer

**DOI:** 10.1371/journal.pone.0017193

**Published:** 2011-02-17

**Authors:** Kai-Yuan Lin, Chein Tai, Jung-Chin Hsu, Chien-Feng Li, Chia-Lang Fang, Hsi-Chin Lai, You-Cheng Hseu, Yi-Feng Lin, Yih-Huei Uen

**Affiliations:** 1 Department of Medical Research, Chi-Mei Medical Center, Tainan, Taiwan; 2 Department of Biotechnology, Chia Nan University of Pharmacy and Science, Tainan, Taiwan; 3 Department of Biotechnology, Southern Taiwan University, Tainan, Taiwan; 4 Department of Pathology, Chi-Mei Medical Center, Tainan, Taiwan; 5 Department of Pathology, Taipei Medical University, Taipei, Taiwan; 6 Department of Pathology, Taipei Municipal Wan Fang Hospital, Taipei, Taiwan; 7 Department of Cosmeceutics, China Medical University, Taichung, Taiwan; 8 Department of Surgery, Chi-Mei Medical Center, Tainan, Taiwan; 9 Department of Electrical Engineering, Southern Taiwan University, Tainan, Taiwan; University of Hong Kong, Hong Kong

## Abstract

**Background:**

Colorectal cancer (CRC) is one of the most common malignancies but the current therapeutic approaches for advanced CRC are less efficient. Thus, novel therapeutic approaches are badly needed. The purpose of this study is to investigate the involvement of nuclear protein kinase CK2 α subunit (CK2α) in tumor progression, and in the prognosis of human CRC.

**Methodology/Principal Findings:**

Expression levels of nuclear CK2α were analyzed in 245 colorectal tissues from patients with CRC by immunohistochemistry, quantitative real-time PCR and Western blot. We correlated the expression levels with clinicopathologic parameters and prognosis in human CRC patients. Overexpression of nuclear CK2α was significantly correlated with depth of invasion, nodal status, American Joint Committee on Cancer (AJCC) staging, degree of differentiation, and perineural invasion. Patients with high expression levels of nuclear CK2α had a significantly poorer overall survival rate compared with patients with low expression levels of nuclear CK2α. In multi-variate Cox regression analysis, overexpression of nuclear CK2α was proven to be an independent prognostic marker for CRC. In addition, DLD-1 human colon cancer cells were employed as a cellular model to study the role of CK2α on cell growth, and the expression of CK2α in DLD-1 cells was inhibited by using siRNA technology. The data indicated that CK2α-specific siRNA treatment resulted in growth inhibition.

**Conclusions/Significance:**

Taken together, overexpression of nuclear CK2α can be a useful marker for predicting the outcome of patients with CRC.

## Introduction

Colorectal cancer (CRC) accounted for about 1 million new cases in 2002 (9.4% of the world total), and unlike most sites, numbers were not so different in men and women (ratio, 1.2∶1) [1]. In terms of incidence, CRC ranks fourth in frequency in men and third in women. There is at least a 25-fold variation in occurrence of CRC worldwide. The highest incidence rates are in North America, Western Europe, and, in men especially, Japan. Incidence tends to be low in Africa and intermediate in southern parts of South America. In Taiwan, CRC ranks as the second most frequently diagnosed malignancy and causes more than 10000 deaths annually (http://www.doh.gov.tw/statistic/index.htm; accessed in December 2008). In spite of the current surgical techniques and chemotherapy that have made significant improvements, the cure rate for advanced CRC remains low and the morbidity remains high [2]. Thus, advances in treatment of this disease are likely to come from a fuller understanding of its pathogenesis and biological features.

Prognosis of newly diagnosed CRC predominantly relies on the American Joint Committee on Cancer (AJCC) stage determined by the depth of invasion, the involvement of the lymph nodes, and distant metastasis [3], [4]. However, in fact, it is well known that patients with the same AJCC stage CRC display survival heterogeneity, with some patients exhibiting relatively short survival times. Accordingly, the identification of more promising prognostic factors that are indeed highly predictive of CRC patients undergoing surgical treatment is mandatory. Many studies have suggested the role that genetic alterations may have in the development and progression of CRC [5], [6]. Molecular pathology may be helpful not only to understand the disease pathogenesis, but also to give useful prognostic molecular markers. Some suggested biological prognostic factors include overexpression of vascular endothelial growth factor (VEGF), enhancer of zeste homologue 2 and transglutaminase 2 [7]–[9].

Protein kinase CK2 (formerly known as casein kinase 2) is a highly conserved serine/threonine kinase. It is distributed ubiquitously in eukaryotic organisms, where it most often appears to exist in tetrameric complexes consisting of two catalytic subunits (αα, α' α' or αα') and two regulatory β subunits [10], [11]. CK2 is a remarkably multifunctional protein kinase with a vast array of more than 300 substrates, many of which are critically involved in the process of cell growth, proliferation, and differentiation [12], [13]. Disruption of *Saccharomyces cerevisiae* genes encoding both CK2 catalytic subunits leads to a failure in development, and the demonstration that knockout of the gene encoding the regulatory CK2 β subunit in mice is also lethal reinforces the importance of CK2 in the maintenance of cell viability in normal cell life and during embryogenesis [14], [15]. In the β subunit, certain cysteine residues may play a role in anchoring the kinase to nuclear structures. CK2 activity may have a role in cell growth through its signaling to key sites in nuclear matrix and chromatin structures [16]. Several growth stimuli can enhance CK2 nuclear shuttling, so that higher nuclear localization is observed in tumor cells compared with normal cells [17], [18].

Moreover, CK2 dysregulation in tumor cells may influence the apoptotic activity and to enhance cell survival [19]. CK2 can exert antiapoptotic effects through various mechanisms. For instance, CK2 counteract apoptosis by protecting Bid from tumor necrosis factor–related apoptosis-inducing ligand (TRAIL)–induced caspase-8–mediated degradation [20]. CK2 is also involved in the phosphorylation of several proteins related to apoptosis, including p53and nuclear factor-κB [21], [22]. In addition, Fas receptor–mediated cell death is regulated by CK2 expression [23].

The level of CK2 seems to be tightly regulated in normal cells, resisting a change in their intrinsic level of CK2 [24]. Increasing evidence indicates that CK2 enzyme is a component of regulatory protein kinase networks that are involved in several aspects of cellular transformation and cancer [25]. Increases in CK2 level and activity have consistently been observed in a variety of human cancers, including mammary gland, head and neck, and kidney cancer [26]–[28]. Overexpression and prognostic significance of CK2 α subunit have only been observed in lung cancer, prostate cancer and leukemia [29]–[31]. To our knowledge, the expression and prognostic significance of nuclear CK2α in human CRC is still unknown. The aims of this study were to investigate the relationship between nuclear CK2α expression and clinicopathologic parameters and prognosis in human CRC patients. We also evaluated the effects of siRNA-inhibited CK2α expression on the proliferation of colon cancer cells.

## Results

### Basic data

Two hundreds and forty-five CRC patients, 139 males and 106 females, were enrolled in this study. Age ranged from 21 to 88 years olds at first diagnosis (mean 64 years). Based on the AJCC classification, there were 36 stage I patients, 98 stage II patients, 110 stage III patients, and 1 stage IV patient. Follow-up for those patients ranged from 2.93 to 123.97 months (mean 68.3 months). During follow-up, 69 patients died of CRC. Postoperative complications did not occur in these patients.

### Nuclear CK2α expression was upregulated and associated with several clinicopathologic parameters in CRC

**Figure 1:**
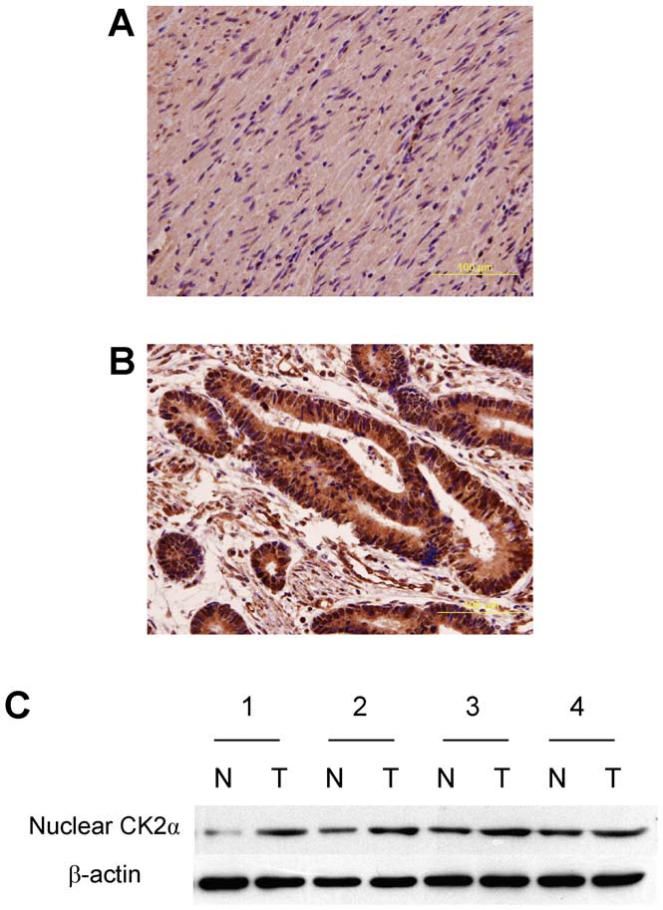
Expression of nuclear CK2α in colorectal tissues. Nuclear CK2α expression in non-tumor and tumor tissues, analyzed by immunohistochemistry, was shown in **Panel A** and **B**, respectively. Magnification: 400×. **Panel C.** Nuclear CK2α expression in four non-tumor/tumor pairs was examined by Western blot.

**Figure 2:**
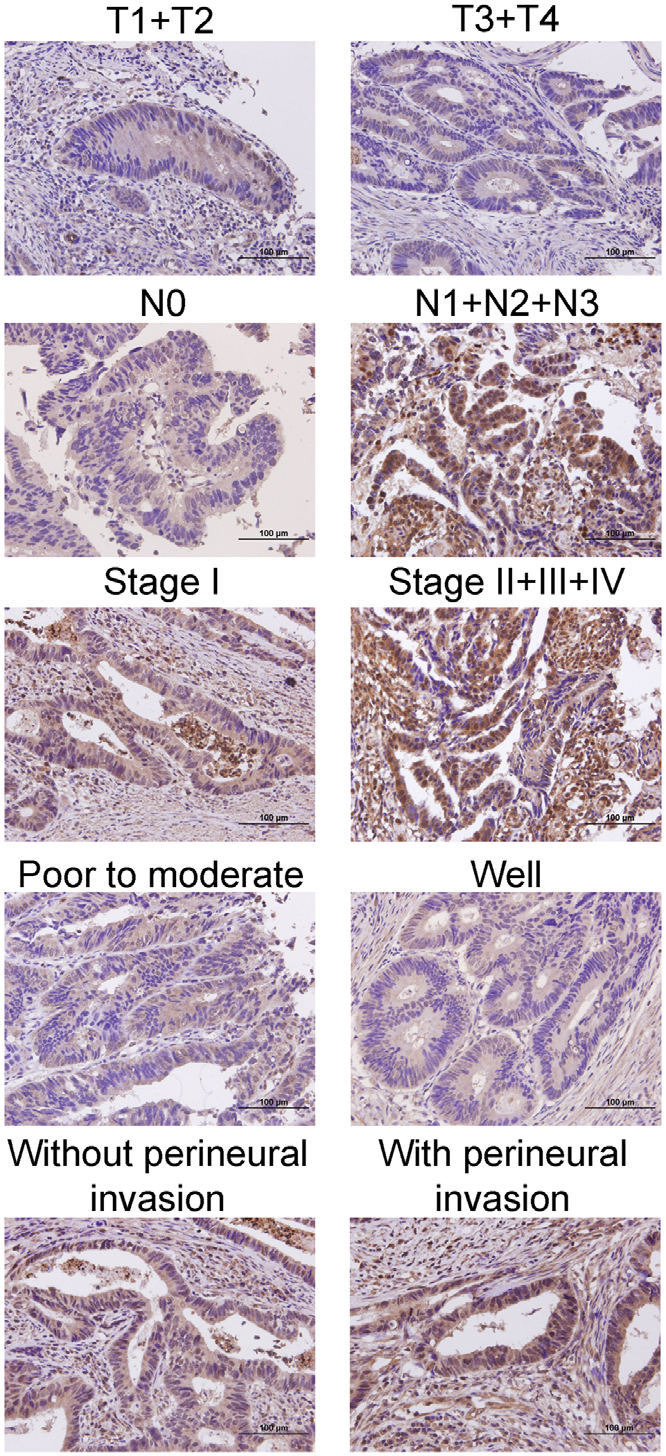
The representative IHC nuclear CK2α staining corresponding to mean nuclear CK2α labeling index values for different parameters. Magnification: 400×.

We investigated the expression of nuclear CK2α in 245 CRC patients by immunohistochemistry, and in four patients by Western blot. The results indicated that nuclear CK2α expression was higher in tumor tissues than in non-tumor tissues (Figure 1). Additionally, quantitative real-time PCR analysis demonstrated that the expression of CK2α was substantially increased in tumor tissues when compared with non-tumor tissues (Table 1). As shown in Table 2, nuclear CK2α expression had a statistically significant correlation with depth of invasion (*P* = 0.008), nodal status (*P*<0.001), AJCC staging (*P*<0.001), degree of differentiation (*P* = 0.011), and perineural invasion (*P* = 0.041). The representative photomicrographs of nuclear CK2α expressions for different parameters were shown in Figure 2. There was no significant association between nuclear CK2α expression and age, gender, and vascular invasion.

**Table 1:** Quantification of CK2α mRNA expression by quantitative real-time PCR in 10 tumor and non-tumor pairs of colorectal tissues.

	Non-tumor			Tumor		
No.	CK2α	β-actin	Δ*C_non-tumor_*	CK2α	β-actin	Δ*C_tumor_*
S0059	33.14	23.37	9.77	32.94	25.01	7.93
S0423	29.67	19.64	10.03	29.01	21.17	7.84
S0475	34.06	22.02	12.04	29.43	19.89	9.54
S0480	35.50	21.35	14.15	31.52	22.03	9.49
S0485	35.68	21.49	14.19	32.87	23.02	9.85
S0597	39.23	24.28	14.95	32.10	19.75	12.35
S0641	30.88	20.29	10.59	32.33	22.45	9.88
S0680	31.95	20.17	11.78	30.94	21.56	9.38
S0706	34.30	21.89	12.41	29.46	20.11	9.35
S0708	30.09	20.93	9.16	33.31	26.04	7.27

**Table 2:** Nuclear CK2α expression in CRC and its correlation with clinicopathologic parameters.

			Mean nuclear CK2α labeling index		
Parameters		n	mean	SD	*P* *
Age	<65y	107	43.32	29.39	0.704
	≥65y	138	44.75	28.94	
Gender	Male	139	43.24	28.80	0.588
	Female	106	45.28	29.55	
Depth of invasion	T1+T2	52	34.62	28.14	0.008
	T3+T4	193	46.68	28.88	
Nodal status	N0	134	34.03	22.28	<0.001
	N1+N2+N3	111	56.31	31.66	
Staging	I	36	24.44	19.38	<0.001
	II+III+IV	209	47.51	29.17	
Differentiation	Poor to moderate	223	45.38	29.42	0.011
	Well	22	31.36	22.21	
Vascular invasion	Absence	196	43.06	29.09	0.256
	Presence	49	48.37	28.97	
Perineural invasion	Absence	214	42.69	29.06	0.041
	Presence	31	54.03	27.73	

*All statistical tests were two-sided. Significance level: *P*<0.05.

### Overexpression of nuclear CK2α as an independent prognostic marker for CRC

**Figure 3:**
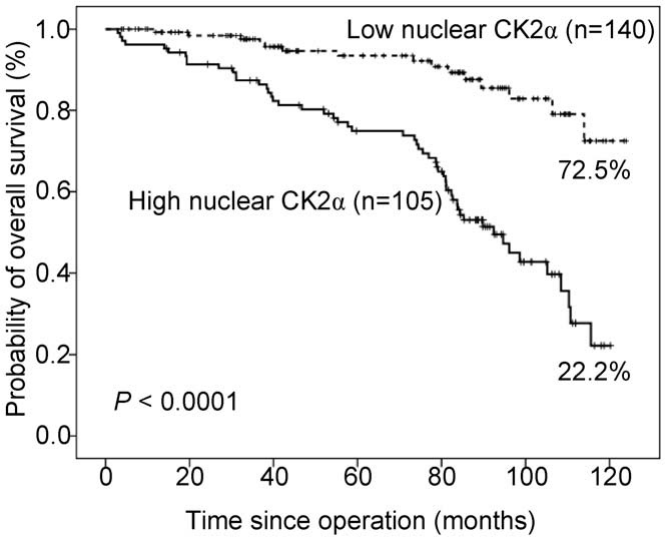
Overall survival analysis of 245 CRC patients stratified by nuclear CK2α immunoreactivity (low nuclear CK2α: mean labeling index ≤40%; high nuclear CK2α: mean labeling index >40%). All statistical tests were two-sided. Significance level: *P*<0.05.

Correlations of clinical outcomes with nuclear CK2α expression are shown in Figure 3. Inferior overall survival was significantly associated with overexpression of nuclear CK2α (mean labeling index >40%, *P*<0.0001). Patients with high expression levels of nuclear CK2α had a ten-year overall survival rate of 22.2% compared with 72.5% for patients with low expression levels of nuclear CK2α.

The uni-variate analysis of prognostic markers of CRC is summarized in Table 3. Nodal status (*P* = 0.025) and overexpression of nuclear CK2α (*P*<0.0001) was correlated significantly with overall survival.

**Table 3:** Uni-variate analysis of prognostic markers in 245 patients with CRC.

Variables		HR	95% CI	*P* *
Depth of invasion	T1+T2	1		
	T3+T4	0.86	0.49–1.50	0.591
Nodal status	N0	1		
	N1+N2+N3	1.74	1.07–2.83	0.025
Staging	I	1		
	II+III+IV	1.10	0.53–2.30	0.803
Differentiation	Well	1		
	Poor to moderate	2.16	0.68–6.88	0.192
Vascular invasion	Absence	1		
	Presence	1.19	0.68–2.09	0.538
Perineural invasion	Absence	1		
	Presence	0.86	0.41–1.79	0.682
Mean nuclear CK2α labeling index	≤40	1		
	>40	4.53	2.55–8.03	<0.0001

*All statistical tests were two-sided. Significance level: *P*<0.05. HR  =  hazard ratio; CI  =  confidence interval.

This association between overexpression of nuclear CK2α and survival remained even after controlling for other well-known prognostic markers in multi-variate analysis (Table 4). In multi-variate analysis, overexpression of nuclear CK2α was prognostically independent (hazard ratio  = 4.53, 95% confidence Interval  = 2.46 to 8.32, *P*<0.0001).

**Table 4:** Multi-variate analysis of prognostic markers in 245 patients with CRC.

Variables		HR	95% CI	*P* *
Nodal status	N0	1		
	N1+N2+N3	1.07	0.60–1.93	0.811
Mean nuclear CK2α labeling index	≤40	1		
	>40	4.53	2.46–8.32	<0.0001

*All statistical tests were two-sided. Significance level: *P*<0.05.

### Effect of knock down of CK2α on colon cancer cell proliferation

**Figure 4:**
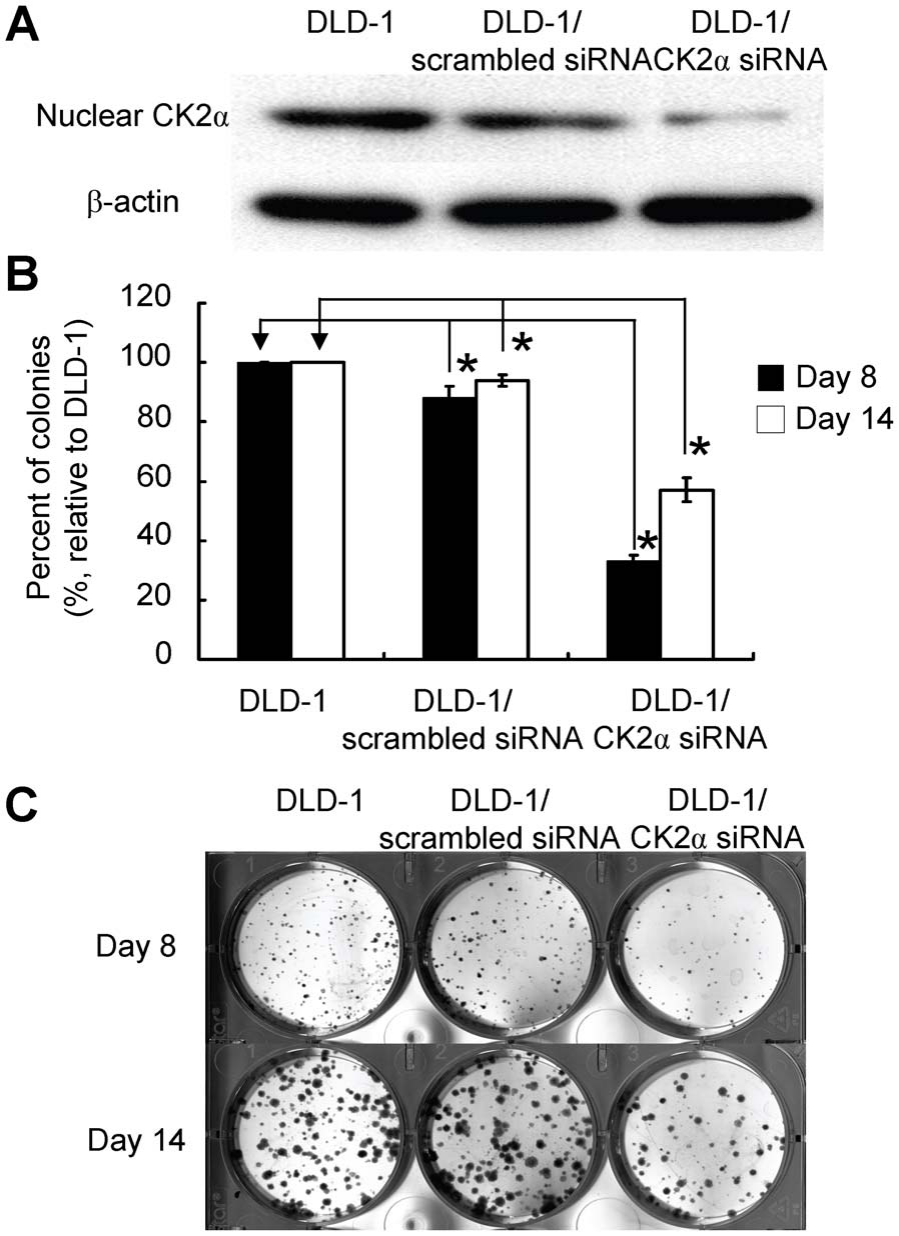
Effect of *CK2*α gene silencing on colon cancer cell proliferation. **Panel A**. Nuclear CK2α expression in siRNA treated and un-treated DLD-1 colon cancer cells was examined by Western blot analysis. **Panel B**. Histograms representing the cell proliferation assay results based on the average of three independent experiments. *denotes *P*<0.001 compared with un-treated DLD-1 cells. **Panel C**. The representative photomicrographs.

To determine the effect of CK2α expression on DLD-1 human colon cancer cell proliferation, *CK2*α gene was knocked down by transfection of CK2α-specific siRNA. Scrambled siRNAs were used as controls. After CK2α-specific siRNA treatment, the expression level of nuclear CK2α in DLD-1 cells was significantly inhibited (Figure 4A). However, scrambled siRNA resulted in no significant inhibition (Figure 4A). We counted the number of colonies of siRNA-treated and un-treated DLD-1 cells. The colonies of CK2α-specific siRNA-treated cells were significantly fewer than those of un-treated and scrambled siRNA-treated cells (decreased to 33% (day 8) and 57% (day 14) of those of un-treated cells, respectively) (Figure 4B). The representative photomicrographs were shown in Figure 4C. The results indicated a role played by CK2α in promoting cell proliferation and is consistent with the clinical data described above.

## Discussion

Elevated CK2α mRNA level has been reported in several human cancers, including melanoma and lung cancer [32], [33]. However, despite these studies, clinical data dealing with the specific expression of CK2α at the protein level are scarce. One recent study showed elevated CK2α protein expression in prostate cancer [30]. To our knowledge, this is the only study using immunohistochemistry for evaluating CK2α expression in human cancers. The expression of nuclear CK2α in human CRC is still unknown. In the present study, the expression levels of nuclear CK2α in colorectal tissues from 245 patients with CRC were assessed, and the results showed that nuclear CK2α expression was higher in tumor colorectal tissues than in non-tumor colorectal tissues.

In addition, our data showed CK2α nuclear accumulation in CRC, consistent with previous study conducted in prostate cancer. The mechanistic basis of CK2α nuclear accumulation is currently unclear but, as a CK2 catalytic subunit, its continuous presence in the nucleus might not only be related to the increased proliferative capacity of dedifferentiated tumour cells but also to their marked resistance to apoptotic signals. What could be the functional consequence of nuclear accumulation of CK2 in cancer cells? One study performed by Scaglioni et al. demonstrated that CK2 phosphorylated the promyelocytic leukemia protein (PML, a tumor suppressor) and targeted it for degradation by the proteasome [34]. Loss of the critical CK2 phosphorylation site in PML resulted in stabilization of this protein, enhancement of PML-induced apoptosis. Moreover, in human non-small cell lung cancers, there is an inverse relationship between PML expression and CK2 activity. Since PML is a nuclear-matrix-associated protein, a nuclear accumulation of CK2 may be functionally relevant to inactivate the tumor-suppressive functions of PML. In another study, CK2 has been recently described as a key regulator of the tumor suppressor NKX3.1 in LNCaP prostate cancer cells. Like PML, it was found that blocking CK2 activity in LNCaP cells with inhibitor apigenin led to a rapid decrease in NKX3.1 accumulation that was rescued by proteasome inhibition [35].

In the current study, we demonstrated for the first time that overexpression of nuclear CK2α in CRC tissues was closely correlated with several clinicopathologic parameters including the depth of tumor invasion, lymph node metastasis, and perineural invasion. Although the mechanisms underlying these associations are still unclear, several lines of evidence for these correlations will be discussed in below. Firstly, the evidence for the association between overexpression of nuclear CK2 and the depth of invasion comes from matrix metalloproteinases (MMPs), which have long been associated with cancer-cell invasion and metastasis [36]. A recent study showed that PC-3 human prostate cancer cells over-expressing membrane type-1 matrix metalloproteinase (MT1-MMP) grew faster than mock-transfected control cells [37]. This result suggested that invasion-promoting MT1-MMP is directly linked to tumor aggressiveness. Genome-wide expression profiling of MT1-MMP-overexpressing versus MT1-MMP-silenced cancer cells and a further data mining analysis of the preexisting expression database of 190 human tumors of 14 cancer types led to identify 11 genes, including CK2, the expression of which correlated firmly and universally with that of MT1-MMP [38]. Secondly, by using immunohistochemistry, the association between VEGF-C and the development of lymph node metastasis has been described by Yonemura Y et al [39]. In response to hypoxia, happened in most tumors grown larger than 1 mm^3^, the expression of VEGF-C can be stimulated by hypoxia-inducible factor-1 (HIF-1) [40]. It was shown that inhibitors of CK2 blocked the activation of HIF-1, and then the expression of VEGF-C [41]. Finally, the increased neurite formation demonstrated in the previous *in vitro* studies suggests that axonal migration may be a key element of perineural invasion. Axonal growth is a complex process that requires neurotrophic growth factors and axonal guidance molecules [42], [43]. One study performed by Arevalo et al. showed that nerve growth factor, one of the neurotrophic growth factors, can stimulate axon growth through activation of CK2 [44].

Our study demonstrated that overexpression of nuclear CK2α can be an independent prognostic marker for CRC. Clinical data dealing with the prognostic value of CK2α are limited. Using global gene expression profiling, the CK2α gene has been identified as a prognostic marker in patients with squamous cell carcinoma of the lung [29]. Laramas et al. provided the evidence for a strong association between a nuclear localization of CK2α and poor prognostic factors in human prostate cancer [30]. In addition, studies of Kim et al. showed that overexpression of CK2α protein in leukemia was associated with poor patient outcome [31]. To our knowledge, there is no report mentioning the prognostic significance of nuclear CK2α in human CRC. It is noteworthy to observe that, for the first time, overexpression of nuclear CK2α can be an independent prognostic marker for CRC. Overexpression of nuclear CK2α is hence a useful marker for predicting the outcome of patients with CRC who had a surgical resection of the tumor. The patients with CRC showing overexpression of nuclear CK2α should be followed-up carefully.

## Materials and Methods

### Study subjects

The patient cohort comprised 245 consecutive CRC cases from 1998 through 2002 documenting pathologic and clinical factors and clinical outcome. None of these patients had received preoperative chemotherapy and/or radiotherapy. The non-tumor part was taken from the grossly normal colorectal mucosa away from the tumor in resected colorectal specimen. Clinicopathologic parameters of CRCs were determined according to the AJCC classification. The follow-up duration was defined as the period between the operation date and day of the last visit, according to the patient's chart. The institutional review board at Chi-Mei Medical Center approved the tissue acquisition protocol to conduct this study. Written informed consent was obtained from each participant before tissue acquisition. Tumor/non-tumor pairs of colorectal tissues were analyzed for nuclear CK2α expression.

### Immunohistochemical analysis

Nuclear CK2α expression was analyzed by immunohistochemistry. Paraffin-embedded tissue blocks were sectioned at 5 µm and transferred to microscope slides (Muto Pure Chemicals, Tokyo, Japan). Hepatoma was used a positive control for CK2α. The negative control consisted of the omission of the primary antibody and incubation with phosphate buffer saline. After rehyfration and antigen retrieval, antigen blocking was performed using Dako REAL Peroxidase-Blocking Solution (Dako, Carpinteria, CA) for 5 minutes. The slides were subsequently incubated with a primary antibody: polyclonal anti-CK2α (Stressgen Bioreagents, Victoria, Canada) for 30 minutes at room temperature at a dilution of 1∶50. Detection of the immunoreactive staining was carried out by the avidin-biotin-peroxidase complex method according to the manufacturer's instructions. A sensitive Dako REAL EnVision Detection System (Dako) was used as the detection system. After incubation with diaminobenzidine for 5-8 minutes, the sections were counterstained and mounted for microscopic interpretation. The immunoreactivity was independently scored by two pathologists (C-F Li and C-L Fang). The percentage of tumor cells with moderate or strong nuclear immunoreactivity was recorded, and scores from the same patient were averaged to obtain a mean nuclear CK2α labeling index. The cutoff of the mean labeling index to define nuclear CK2α overexpression was nuclear reactivity in >40% cells (median value). Clinical data collection and immunohistochemical analysis were performed independently of each other, in an investigator-blinded study.

### RNA extraction and cDNA synthesis

According to the manufacturer's instructions, total RNA from 10 tumor and non-tumor pairs of colorectal tissues was isolated by using an RNA extraction kit (Sigma, St. Louis, MO). RNA quality was analyzed by using Agilant 2100 Bioanalyzer. The RIN values of all 20 samples were above 7. cDNA synthesis was performed as described in our previous study [45]. Synthesized cDNA was stored at −20°C until use.

### Primers and probes

Taqman Gene Expression Assays including primers and probes of CK2α and β-actin, an internal control, were purchased from Applied Biosystems. The Assay numbers of CK2α and β-actin were Hs00751002_s1, and Hs99999903_m1, respectively.

### Quantitative real-time PCR

The expression levels of the target genes were measured using quantitative real-time PCR in the ABI Prism 7300 Sequence Detection System (Applied Biosystems) as described in our previous study [45]. Threshold cycle (*C_t_*) is the fractional cycle number at which the fluorescence generated by cleavage of the probe exceeds a fixed level above baseline. For a chosen threshold, a smaller starting copy number results in a higher *C_t_* value. The amount of CK2α mRNA in tumor or non-tumor tissues, standardized against the amount of β-actin mRNA, was expressed as Δ*C_tumor_* or Δ*C_non-tumor_*  = *C_t_*_(CK2α)_ - *C_t_*_(β-actin)_.

### Cell culture and siRNA treatment

Human colon cancer cell line (DLD-1) was obtained from the Food Industry Research and Development Institute (Hsinchu, Taiwan). Cells were cultured in RPMI-1640 supplemented with 10% heat-inactivated fetal bovine serum (Moregate BioTech, Bulimba QLD, Australia), 100 units/ml penicillin G, 100 µg/ml streptomycin sulfate, and 250 ng/ml amphotericin B (all from Sigma). Cells were grown at 37°C in a humidified atmosphere containing 5% CO_2_. For siRNA treatment, cells were transfected with 30 nM CK2α-specific or scrambled siRNA (Applied Biosystems), using siPORT NeoFX Transfection Agent. 48 hours post-transfection, nuclear proteins were extracted, and then Western blot was performed to evaluate the effect of siRNA treatment.

### Nuclear protein preparation

Total cellular and tissue nuclear proteins were extracted with NE-PER Nuclear and Cytoplasmic Extraction Reagents (Pierce Biotechnology, Rockford, IL), according to the instructions of the manufacturer. The samples were stored at −80°C until used. The protein concentration was determined using a BCA Protein Assay Kit (Pierce Biotechnology) with bovine serum albumin as a standard.

### Immunoblotting

Denatured protein samples were subjected to 10% SDS-PAGE. Proteins were transferred to nitrocellulose membranes. Blocked blots were incubated at room temperature overnight with anti-CK2α polyclonal antibody (1∶161 dilution). β-actin (Sigma, 1∶8000 dilution) was used as an internal control for equal protein loading. Blots were further incubated with secondary antibodies conjugated with peroxidase (Sigma) for 1 hour at room temperature. Enhanced chemiluminescence reagents (Pierce Biotechnology) were used to visualize the targeted proteins, which were then semi-quantitatively measured by densitometry. All the experiments were conducted three times, independently.

### Cell proliferation assay

After siRNA treatment, the transfected cells were cultured in a 37°C, 5% CO_2_ incubator for 4, 8 and 14 days. Individual colonies (>50 cells/colony) were fixed, stained using a solution of 1% crystal violet in methanol for 30 minutes, and counted. The assay was conducted three times, independently. For each experiment, two wells per condition were used. Error bars are S. D.

### Statistical analysis

Differences in nuclear CK2α expression between tumor and non-tumor tissues in the same patient and in cell proliferation were analyzed using a paired *t* test. Some clinicopathologic parameters were examined in this study. They were age, gender, depth of invasion, nodal status, staging, degree of differentiation, vascular and perineural invasion. The correlation between nuclear CK2α expression and clinicopathologic parameters was examined with χ^2^ test. All time-to-event endpoints according to various clinicopathologic parameters were plotted by the Kaplan-Meier method and the significance was then determined by a uni-variate log-rank test. In principle, those significant parameters in uni-variate analysis (*P*≤0.05) were entered into multi-variate Cox regression model to determine the hazard ratio and independence of prognostic impact in a stepwise backward fashion. All of the data were analyzed using the SPSS software version 14 (SPSS, Chicago, IL). A *P* value of <0.05 was considered significant.
